# Differences in healthcare visit frequency and type one year prior to stroke among young versus middle-aged adults

**DOI:** 10.1186/s12913-021-06064-5

**Published:** 2021-01-22

**Authors:** Brandi L. Vollmer, Xing Chen, Erin R. Kulick, Mitchell S. V. Elkind, Amelia K. Boehme

**Affiliations:** 1 Department of Neurology, Vagelos College of Physicians and Surgeons, Columbia University, New York City, NY USA; 2 Department of Epidemiology, Mailman School of Public Health, Columbia University, New York, NY USA; 3 Department of Biostatistics, Mailman School of Public Health, Columbia University, New York, NY USA; 4 Department of Epidemiology and Biostatistics, Temple University College of Public Health, Temple University, Philadelphia, PA USA; 5 Division of Neurology Clinical Outcomes Research and Population Sciences, Columbia University, 710 West 168th Street, Room 642, New York, NY 10032 USA

**Keywords:** Stroke, Stroke in the young, Risk factors, Healthcare utilization

## Abstract

**Background:**

The incidence and prevalence of stroke among the young are increasing in the US. Data on healthcare utilization prior to stroke is limited. We hypothesized those < 45 years were less likely than those 45–65 years old to utilize healthcare in the 1 year prior to stroke.

**Methods:**

Patients 18–65 years old who had a stroke between 2008 and 2013 in MarketScan Commercial Claims and Encounters Databases were included. We used descriptive statistics and logistic regression to examine healthcare utilization and risk factors between age groups 18–44 and 45–65 years. Healthcare utilization was categorized by visit type (no visits, inpatient visits only, emergency department visits only, outpatient patient visits only, or a combination of inpatient, outpatient or emergency department visits) during the year prior to stroke hospitalization.

**Results:**

Of those 18–44 years old, 14.1% had no visits in the year prior to stroke compared to 11.2% of individuals aged 45–65 [OR = 1.30 (95% CI 1.25,1.35)]. Patients 18–44 years old had higher odds of having preventive care procedures associated with an outpatient visit and lower odds of having cardiovascular procedures compared to patients aged 45–65 years. Of stroke patients aged 18–45 and 45–65 years, 16.8 and 13.2% respectively had no known risk for stroke.

**Conclusions:**

Patients aged 45–65 were less commonly seeking preventive care and appeared to be seeking care to manage existing conditions more than patients aged 18–44 years. However, as greater than 10% of both age groups had no prior risk, further exploration of potential risk factors is needed.

**Supplementary Information:**

The online version contains supplementary material available at 10.1186/s12913-021-06064-5.

## Background

With 795,000 strokes occurring in the US annually, stroke is currently the leading cause for long-term adult disability and ranked fifth in cause of death [1–5]. Although mortality has decreased over time, high stroke-associated morbidity continues to be a significant burden with approximately $17.5 billion per year for direct stroke costs [4, 5]. Importantly, the incidence and prevalence of stroke among the young (18 to 44 years old) is increasing in the US, with approximately 10–14% of ischemic strokes occurring in this age group [6]. Evidence suggests this increase is not due to improved imaging techniques, but coincides with increasing traditional risk factors among those hospitalized [7].

Reducing the burden of stroke in the young population requires identification of modifiable risk factors. However, among all age groups conventional risk factors only account for 80% of risk associated with incident events, leaving a substantial proportion of risk unexplained [8]. Moreover, risk factors in the young differ from those in older populations. Previously identified stroke in the young risk factors include hypercoagulable state, nephrotic syndrome, renal disease, migraine, valvular heart disease, congenital heart disease and patent foramen ovale. Recent research has also recognized infections as a novel risk factor for stroke, particularly in young adults [9–13]. Vascular inflammation in response to an infection may promote coagulation, which then may increase risk of stroke, and as aging contributes to a decline in immune function, this potential mechanism of stroke may be more common in younger individuals [14, 15]. Additionally, younger populations with high psychosocial burden or history of physical trauma or cancer events have an increased risk of stroke [16–18].

However, despite emerging evidence for novel risk factors, there are limited studies examining management of these conditions. Further, prior health statistics have shown, in the general population, that those aged 18–44 to have lower healthcare utilization compared to those aged 45–64 [19]. However, stroke patients are distinct from the general population due to a difference in disease burden, and disease burden plausibly influences the likelihood of utilizing healthcare systems. To date, no study has investigated healthcare system utilization prior to a stroke event, particularly in younger patients. Understanding healthcare system utilization and risk factors in this population may allow for better prevention strategies and knowledge of areas in need of further research. Therefore, through a retrospective cohort study design, we addressed this gap in knowledge though assessment of care-seeking patterns 1 year prior to stroke in young (18–44 years) compared to middle aged (45–65 years) populations. We hypothesized that a higher proportion of patients aged 18–44 with stroke had no healthcare visit in the 1 year prior to stroke than those 45–65 years. We assessed this hypothesis through examination of preceding visit types, if any, during the 1 year prior to stroke hospitalization, procedures associated with these visits, and history of risk factors at the time of stroke hospitalization, which may influence healthcare utilization.

## Methods

### Data source and study patients

We obtained patient data for our study from the MarketScan Commercial Claims and Encounters database, an administrative dataset containing longitudinal data for approximately 230 million de-identified patients. MarketScan contains data from patients enrolled in employee-sponsored insurance programs. Data is collected, de-identified and standardized, and includes demographic characteristics as well as International Classification of Diseases, 9th Revision (ICD-9) diagnosis and procedure codes for all inpatient, outpatient, and emergency department (ED) visits. Each patient is given a de-identified patient code to allow for linking of data and tracking over time. For this study, patients who had a stroke between 2008 and 2014 while enrolled in MarketScan and those age 65 or younger were included for analysis. Our study was limited to the period between 2008 and 2014 because of licensing and cost constraints related to obtaining access to MarketScan data at our institution. We categorized patients by their age at first stroke, 18–44 and 45–65 years. This study was approved by the institutional review board at Columbia University Medical Center; the need for review was waived as data used were publicly available and did not contain direct personal identifiers.

### Outcome measures

We selected patients with ischemic strokes and intracerebral hemorrhagic strokes defined using ICD-9 codes 433.× 1 (where “x” can vary by specify arterial distribution), 434.00, 434 (excluding 434.× 0 with exception of 434.00), 436 and 431. Subarachnoid hemorrhagic strokes were excluded from this analysis as they are largely dependent on experiencing trauma or aneurysm, rather than risk factors examined in our study [20].

### Exposure measures

Additional Table 1 presents ICD-9 codes used for identification of healthcare utilization types and risk factors. Age groups were defined as 18–44 years and 45–65 years at time of stroke. We categorized individuals by preceding visit types during the 1 year prior to stroke hospitalization; no prior visits, ED visits only, inpatient visits only, outpatient patient visits only, and a combination of inpatient, outpatient or emergency department visits. Due to small sample sizes, detailed descriptions of patients with inpatient visits only (0.1% of total patients included in our study) and ED visits only (1.3% of total patients included in our study) are not presented. Additionally, we categorized outpatient visits based on associated MarketScan procedure group codes of interest including preventive care, vaccination, cardiovascular, neurology, chiropractic, or emergent office visit to calculate percentage of patients with at least one prior visit for these categories in the year preceding stroke. MarketScan procedure group codes are groups of related outpatient procedures, based on Current Procedural Terminology, 4th Edition, ICD-9-CM, or HCPCS procedure codes [21]. MarketScan procedure group codes for preventative care includes physical exams, counseling/guidance/risk factor reduction, and ordering of laboratory/diagnostic procedures. All immunizations were classified as vaccinations and not as preventive care. Cardiovascular procedures included, but are not limited to, EKGs and echocardiograms. Procedure group codes used for categorization of outpatient visits are presented in Additional Table 1. For categorization of risk groups, we collected medical history at the time of stroke hospitalization using ICD-9 codes. Risk groups were categorized as 1) metabolic causes; 2) infections; 3) stroke in the young (SITY) risk factors; 4) substance use history; 5) vascular disease history; 6) trauma and/or cancer. Additional Table 2 includes detailed definitions of risk groups.

### Statistical analysis

For each age group, we examined the proportions as n (%) who met criteria for each visit type, including those with ED visits only, inpatient visits only, outpatient visits, only, or had some combination of inpatient, outpatient and/or ED visits in the year prior to stroke. Additionally, we examined distributions of demographic characteristics, medical history assessed at time of stroke, and risk groups as mean [standard deviation (SD)] for continuous variables and proportions as n (%) for categorical variables by visit types prior to stroke (no visits, outpatient visits only, combination of visits). For total number of visits prior to stroke and days from last visit, we also examined median [interquartile range (IQR)]. We calculated odds ratios (ORs) and 95% confidence intervals (95% CIs) using logistic regression comparing those aged 18–44 years to those aged 45–65 years for odds of having no visit in the year prior to stroke and odds of meeting criteria for each risk group. Additionally, for those with outpatient visits only and those with a combination of visits, odds ratios (ORs) and 95% confidence intervals (95% CIs) were calculated comparing those aged 18–44 years to those aged 45–65 years to assess odds of having select procedure codes.

## Results

We identified a total of 169,358 patients with incident stroke for inclusion in this study, consisting of 24,103 patients between ages 18–44 and 145,255 ages 45–65 years. Of those 18–44 years of age, 14.1% had no visits in the year prior to stroke compared to 11.2% of individuals aged 45–65. Those aged 18–44 had 1.30 times the odds of having no visit in the year prior to stroke compared to 45–65 (95% CI 1.25,1.35) year-olds. Of those 18–44 years of age, 516 (2.1%) had only ED visits, 28 (0.1%) had only inpatient visits, 10,812 (44.9%) had only outpatient visits, and 9356 (38.8%) had some combination of inpatient, outpatient and/or ED visits in the year prior to stroke. Of those 45–65 years of age, 1649 (1.1%) had only ED visits, 113 (0.1%) only had inpatient visits, 76,226 (52.5%) only had outpatient visits and 50,969 (35.1%) had a combination of inpatient, outpatient and ED visits in the year prior to stroke.

For those 18–44 years of age, 36.9% of those with no visits, 53.3% of those with outpatient visits only and 59.1% of those with a combination of visits were female (Table 1). For those 45–65 years of age, 33.3% of those with no visits, 42.6% of those with outpatient visits only and 47.5% of those with a combination of visits were female. For both age groups, those with a combination of visit types had the largest number of total visits and the shortest time since last visit prior to stroke. For those 18–44 years of age, the median time from last visit for those with at least one visit in the year prior to stroke was 6 (IQR: 1–35) days. The median time from last visit to stroke in those 18–44 years was 13 (IQR: 2–65) days for those with only outpatient visits and 3 (IQR: 1–14) days for a combination of visits. For those 45–65 years of age, the median time from last visit for those with at least one visit in the year prior to stroke was 6 (IQR: 2–27) days, 10 (IQR: 2–45) days for those with only outpatient visits and 4 (IQR: 1–12) days for a combination of visits.

**Table 1:** Demographics and prior history assessed at time of stroke by age group and visit type

	Ages 18–44						Ages 45–65					
	No Visits Prior to Stroke (*N* = 3391)		Outpatient Visits Only (*N* = 10,812)		Combination^a^ of Visits (*N* = 9872)		No Visits Prior to Stroke (*N* = 16,298)		Outpatient Visits Only (*N* = 76,226)		Combination^a^ of Visits (*N* = 52,618)	
**Age** (Mean, SD)	36.5	7.0	36.5	6.8	35.8	7.1	55.8	5.4	57.1	5.2	56.8	5.3
**Sex – Female** (n, %)	1252	36.9%	5760	53.3%	5831	59.1%	5428	33.3%	32,437	42.6%	25,003	47.5%
**Total # of Visits Prior**												
Mean, SD	–	–	7.8	10.9	20.8	24.6	–	–	10.8	13.2	26.3	27.6
Median, IQR	–	–	4	2–9	13	6–25	–	–	7	3–14	18	9–34
**Days from Last Visit**												
Mean, SD	–	–	49.4	75.1	19.6	43.1	–	–	39.5	65.4	16.1	37.0
Median, IQR	–	–	13	2–65	3	1–14	–	–	10	2–45	4	1–12
**Medical History** (n, %)^b^												
Diabetes	267	7.9%	757	7.0%	888	9.0%	2645	16.2%	14,612	19.2%	11,122	21.1%
Hypertension	1347	39.7%	3222	29.8%	2945	29.8%	10,446	64.1%	43,782	57.4%	27,409	52.1%
Obesity	126	3.7%	326	3.0%	352	3.6%	460	2.8%	2115	2.8%	1551	2.9%
Infections	393	11.6%	1020	9.4%	1271	12.9%	1845	11.3%	7038	9.2%	6835	13.0%
Coagulopathy	159	4.7%	731	6.8%	741	7.5%	460	2.8%	2182	2.9%	2237	4.3%
Hypercoagulable state	77	2.3%	429	4.0%	420	4.3%	109	0.7%	741	1.0%	775	1.5%
Migraine	281	8.3%	1232	11.4%	1207	12.2%	324	2.0%	2348	3.1%	1793	3.4%
Valvular heart disease	356	10.5%	1218	11.3%	1069	10.8%	2149	13.2%	9205	12.1%	6004	11.4%
Congenital Heart Disease	6	0.2%	33	0.3%	22	0.2%	5	0.0%	41	0.1%	28	0.1%
Patent foramen ovale	268	7.9%	906	8.4%	1716	17.4%	501	3.1%	2349	3.1%	1211	2.3%
Alcohol abuse	607	17.9%	1279	11.8%	1084	11.0%	3801	23.3%	11,584	15.2%	6823	13.0%
Drug Abuse/Dependence	183	5.4%	294	2.7%	258	2.6%	876	5.4%	1678	2.2%	1209	2.3%
Smoking	515	15.2%	1130	10.5%	954	9.7%	3372	20.7%	10,604	13.9%	6041	11.5%
Trauma	170	5.0%	346	3.2%	259	2.6%	287	1.8%	910	1.2%	600	1.1%

^a^ including inpatient, outpatient and/or emergency department

^b^ Collected at time of stroke

Compared to those aged 45–65 years, those aged 18–44 years with outpatient visits only had increased odds of having a preventive care [OR = 1.36 (95% CI: 1.30, 1.43)], chiropractic [OR = 1.28 (95% CI: 1.19, 1.38)] or emergent office [OR = 1.96 (95% CI: 1.71, 2.25)] procedure code associated with a visit and decreased odds of having a vaccination [OR = 0.61 (95% CI: 0.57, 0.65)], cardiovascular [OR = 0.40 (95% CI: 0.38, 0.42)] or neurologic [OR = 0.85 (95% CI: 0.77, 0.92)] procedure (Table 2). Results were similar when examining procedures among those with a combination of visits, with the exception of neurologic procedures. For those with a combination of visits, those aged 18–44 were more likely to have a neurologic [OR = 1.08 (95% CI: 1.02, 1.15)] procedure compared to those aged 45–55 years.

**Table 2:** Visit type by age and visit group

	Outpatient Visits Only						Combination^a^ of Visits					
	Ages 18–44 (*N* = 6740)		Ages 45–65 (*N* = 80,298)		Odds Ratio^b^	95% CI	Ages 18–44 (*N* = 6555)		Ages 45–65 (*N* = 55,935)		Odds Ratio^b^	95% CI
Visit type	N	%	N	%			N	%	N	%		
Preventive care	2935	27.1%	16,363	21.5%	1.36	1.30, 1.43	2258	22.9%	9483	18.0%	1.39	1.32, 1.47
Vaccination	1223	11.3%	13,138	17.2%	0.61	0.57, 0.65	1438	14.6%	11,360	21.6%	0.63	0.59, 0.67
Cardiovascular	2617	24.2%	33,791	44.3%	0.40	0.38, 0.42	5826	59.0%	40,467	76.9%	0.43	0.41, 0.45
Neurology	555	5.1%	4613	6.1%	0.84	0.77, 0.92	1409	14.3%	7148	13.6%	1.08	1.02, 1.15
Chiropractic	823	7.6%	4611	6.0%	1.28	1.19, 1.38	676	6.8%	3160	6.0%	1.18	1.08, 1.29
Emergent office	266	2.5%	968	1.3%	1.96	1.71, 2.25	282	2.9%	910	1.7%	1.70	1.49, 1.95

Note: As procedure codes are associated with inpatient, outpatient or emergency department visits, those with no visits prior to stroke had no procedure codes for analysis

^a^ including inpatient, outpatient and/or emergency department

^b^ Ages 18–44 years compared to ages 45–65 years

**Fig. 1:**
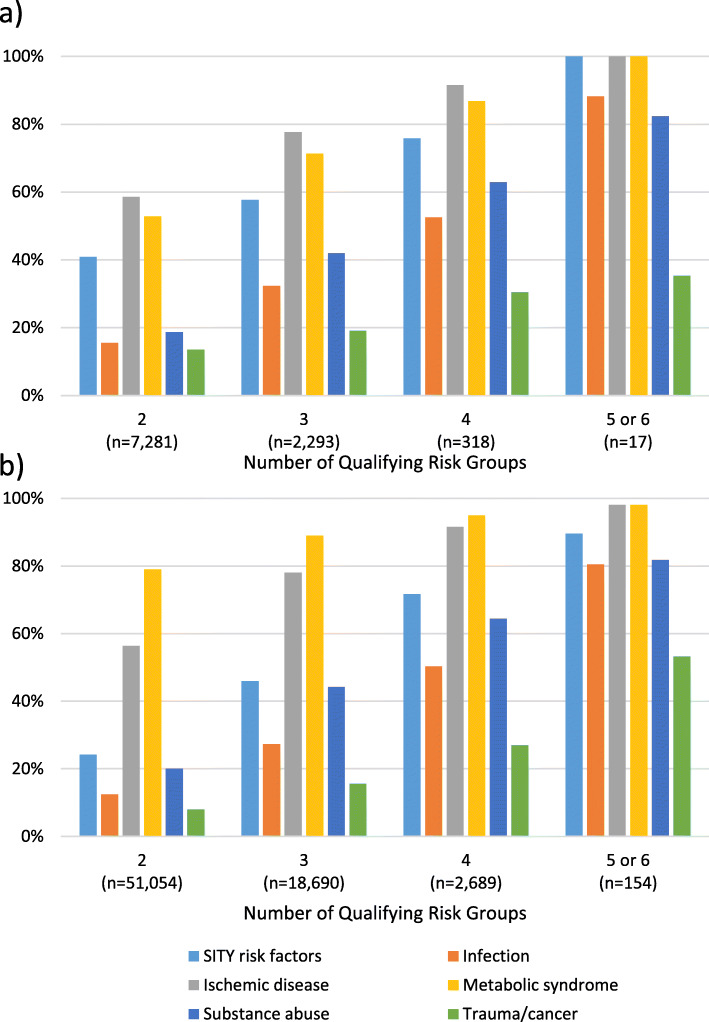
The percentages of patients who meet the criteria for each risk group by the number of risk groups for which they qualify in those a) 18–44 years old, and b) 45–65 years old

When compared to 45–65 year-olds, those 18–44 years of age had higher odds of having SITY risk factors [OR = 1.85 (95% CI: 1.79, 1.90)], trauma or cancer [OR = 1.15 (95% CI: 1.09, 1.21)], or no known risk factors [OR = 1.33 (95% CI: 1.28, 1.38)] at the time of stroke. Those 18–44 years of age had lower odds of having metabolic syndrome [OR = 0.34 (95% CI: 0.33, 0.34)] or substance use [OR = 0.79 (95% CI: 0.76, 0.82)] compared to those 45–65 years old (Table 3). Odds of having infections or ischemic disease were similar between the age groups. When examining the percentage of patients who met risk group criteria by age group and visit type, those with no visits prior to stroke had the highest percentage who met the criteria for multiple risk groups for both those 18–44 years of age (47.0%) and those 45–65 year of age (55.2%) (Fig. 1 and Table 4).

**Table 3:** Risk groups for stroke by age

	Ages 18–44 (*n* = 24,103)		Ages 45–65 (*n* = 145,255)		Odds Ratio^a^	95% CI
Risk group	N	%	N	%		
Metabolic Syndrome	8402	34.9%	89,322	61.5%	0.34	0.33, 0.34
Infections	2619	10.9%	15,520	10.7%	1.02	0.98, 1.07
SITY risk factors	7511	31.2%	28,596	19.7%	1.85	1.79, 1.90
Substance Use	3156	13.1%	23,213	16.0%	0.79	0.76, 0.82
Vascular Disease	9461	39.3%	57,056	39.3%	1.00	0.97, 1.03
Trauma or Cancer	1797	7.5%	9500	6.5%	1.15	1.09, 1.21
No prior risk	4047	16.8%	19,167	13.2%	1.33	1.28, 1.38

*SITY* Stroke in the young

^a^ Ages 18–44 years compared to ages 45–65 years

**Table 4:** Risk groups by age and visit group

	No Visits Prior to Stroke				Outpatient Visits Only				Combination^a^ of Visits			
	Ages 18–44 (*N* = 3391)		Ages 45–65 (*N* = 16,298)		Ages 18–44 (*N* = 10,812)		Ages 45–65 (*N* = 76,226)		Ages 18–44 (*N* = 9872)		Ages 45–65 (*N* = 52,618)	
Risk Group	N	%	N	%	N	%	N	%	N	%	N	%
Multiple groups	1594	47.0%	9002	55.2%	4172	38.6%	36,579	48.0%	4131	41.8%	26,943	51.2%
SITY risk factors only	335	9.9%	523	3.2%	1463	13.5%	3164	4.2%	1156	11.7%	1899	3.6%
Infections only	54	1.6%	238	1.5%	225	2.1%	1099	1.4%	282	2.9%	1293	2.5%
Vascular disease only	387	11.4%	1070	6.6%	1493	13.8%	5875	7.7%	1219	12.3%	4139	7.9%
Metabolic syndromes only	397	11.7%	3231	19.8%	1131	10.5%	16,805	22.0%	1097	11.1%	9583	18.2%
Substance abuse only	117	3.5%	456	2.8%	288	2.7%	1572	2.1%	213	2.2%	847	1.6%
Trauma/cancer only	23	0.7%	55	0.3%	94	0.9%	644	0.8%	158	1.6%	975	1.9%
No groups at all	484	14.3%	1723	10.6%	1946	18.0%	10,488	13.8%	1616	16.4%	6939	13.2%

*SITY* Stroke in the young

^a^ including inpatient, outpatient and/or emergency department

## Discussion

Our retrospective cohort study using MarketScan data demonstrates commercially insured individuals aged 18–44 years had greater odds of having no inpatient, outpatient or ED visit 1 year prior to stroke compared to those aged 45–65 years. Of those with outpatient or a combination of visits, younger patients had increased odds of having a preventative care visit, but lower odds of having a vaccination compared to older patients. Of all patients who had a visit, half had a visit within 6 days prior to stroke. Importantly, 16.8% of stroke patients aged 18–44 and 13.2% of those 45–65 years had no known risk factor for stroke.

Health statistics have previously shown those aged 18–44 have lower healthcare utilization, with 21.9% not having had contact with their physician within the past year compared to 13.6% of those aged 45–64 [19]. Similarly, the number of outpatient visits per 100 person-years in the general population increases with age group [22]. Therefore, although statistically significant, it may be surprising that we did not see a larger numerical difference between the percentage of young and older age groups with no visits prior to stroke (14.1% vs 11.2%). Consistent with previously described trends, there also appears to be an increase in median number of visits for the older age group for those with outpatient visits only (4 vs 7) or a combination of visits (13 vs 18). These smaller differences seen in healthcare utilization may be due to differences in study populations, as this study investigates a privately insured population who might be more likely to seek regular care. Additionally, those who have experienced a stroke may have greater morbidity and risk factors than the general population, particularly among younger age groups, hence increasing the likelihood of seeking care. However, cost of healthcare, type of insurance coverage, and length of insurance coverage may also play a role [23]. Further, prior experience could influence the degree to which someone attends preventative clinic visits, as people want to have a caring provider who they feel comfortable enough with to express their concerns [24].

In both age groups of our study, a nominally greater proportion of females had outpatient visits or a combination of visits than no visits at all (Table 1). This is supportive of previous literature demonstrating greater healthcare utilization among females [19, 22]. Interestingly, our younger age group of those who experienced stroke consisted of more females than males (53.3% vs 46.7%). While this was not expected, as men have been shown to have increased incidence rates of stroke compared to women, particularly in younger ages [25], our study did not investigate incidence rates. This difference in proportions may be due to characteristics of the population enrolled in MarketScan.

When examining procedure codes associated with an outpatient visit, those aged 18–44 years had increased odds of seeking preventive care procedures than those 45–65 years. While this may not be expected based on prior research indicating decreased healthcare utilization in younger age groups, this is likely driven by differences in comorbidities [22]. Instead of seeking preventive care, those aged 45–65 are likely seeking care to manage current conditions. This is demonstrated by increased metabolic syndrome in those 45–65 compared to 18–44 years and additionally increased cardiovascular visits in the year prior to stroke.

However, there was decreased utilization of vaccines among younger populations. While these age differences support existing literature, the percentage of patients who had a vaccine within the year prior to stroke is concerning when considering the need for an annual flu vaccine. Our study captures all types of vaccinations administered at an inpatient, outpatient or emergency department visit, including, but not limited to, the flu vaccine. However, vaccine utilization for all immunizations in our study, ranging from 11.3 to 21.5% depending on age group and visit type, are well below annual estimates of flu vaccine utilization from the CDC. For the 2018–2019 flu year, the CDC estimated a vaccination coverage of 34.9% of 18–49 year olds and 47.3% of 50–64 year olds [26]. This may in part be due to individuals receiving flu vaccinations from sources that were not captured within the MarketScan dataset, such as through a pharmacy or work program. However, a previous meta-analysis found those vaccinated against the flu had a decreased risk of developing stroke [27], thus lower vaccination rates among our study population may be expected. As it is currently unclear if vaccination is a marker for health care utilization, resulting in a reduction in stroke risk due to management of risk factors rather than the influenza vaccination itself, future studies should thoroughly examine differences in vaccination rates among those who do and do not utilize healthcare among at risk populations.

When evaluating risk groups for both age groups, results indicate those with no visits 1 year prior had the highest proportions of patients with prior risk for stroke. A higher percentage of patients with no visits prior to stroke had prior history of hypertension, alcohol abuse, drug abuse/dependence, and smoking than all other visit groups for both age groups. Likely, despite having private insurance, these patients are not reached by current clinical interventions to reduce or manage risk factors, illustrating the need for population-based prevention methods.

Not surprisingly, the greatest proportions of stroke patients for each visit type were included in multiple risk groups. Modifiable or manageable risk factors, particularly hypertension, were common suggesting a large portion of strokes may have been preventable. However, it is concerning that 16.8 and 13.2% of those aged 18–44 and 45–65 had no prior risk as seen in Table 4. This could in part be due to limited detection of risk factors as diagnostic tools are continuously being developed, [28] Further, under reporting in this administrative dataset may occur, particularly for conditions that do not contribute to reimbursement, as for example, low prevalence of obesity was observed for both age groups (ranging from 2.7 to 3.7%). However, additional research is likely needed to identify novel risk factors, further informing stroke mechanism and areas for prevention in the younger population, as modifiable risk factors including hypertension, smoking and alcohol abuse were less common in those aged 18–44 years.

This study has some limitations. Medical history and risk factors may not be readily captured in MarketScan as it is an administrative database, thus prevalence estimates may be underestimated in our study. Additionally, assessed ICD-9 procedure codes are associated with outpatient visits. As patients may seek care through additional sources, such as through work programs or a pharmacy, our results likely underestimate procedure, particularly vaccination coverage. Finally, MarketScan is a dataset of insurance claims and does not capture un-insured individuals or individuals with insurances that do not participate in MarketScan. However, as this is a descriptive study including a large sample size representative of a national population, we believe our study can inform additional questions pertaining to healthcare utilization and risk factor prevalence among young stroke patients.

## Conclusions

In conclusion, no prior study had investigated healthcare system utilization prior to a stroke event, particularly in younger patients, though understanding healthcare utilization in this population can inform prevention strategies, such as risk factor management. Our retrospective cohort study including a commercially insured sample aimed to address this gap and found those aged 18–44 years had increased odds of having no inpatient, outpatient or ED visits 1 year prior to stroke compared to those aged 45–65 years. Those with no prior visits accounted for 14.1 and 11.2% of 18–44 and 45–65 year-olds, respectively. This difference between age groups is smaller in our study than the general population, highlighting how our population is distinct. While older patients aged 45–65 years were less commonly seeking preventive care, they had a higher median number of visits and appeared to be seeking care to manage existing conditions more so than patients aged 18–44 years. As vaccine utilization for all immunizations in our study were low, future studies should closely examine differences in vaccination rates among those who do and do not utilize healthcare among at risk populations to determine the relationship between vaccination and stroke risk. Additionally, our study describes the need for population-based interventions to lower modifiable risk factors as a higher percentage of patients with no visits prior to stroke had prior history of hypertension, alcohol abuse, drug abuse/dependence, and smoking for both age groups. However, as greater than 10% of those aged 18–44 and 45–65 years had no known prior risk, further exploration of novel risk factors is needed.

## Supplementary Information

**Additional file 1: Table 1.** International Classification of Diseases, 9th Revision, Clinical Modification Codes Used to Identify Outcome or covariates of interest and MarketScan Procedure Group Codes. **Table 2.** Definitions used for classifying risk groups.

## Data Availability

The datasets generated and analyzed during the current study are available in the MarketScan Commercial Claims and Encounters database.

## Abbreviations

ICD-9: International Classification of Diseases, 9th Revision
ED: Emergency department
SD: Standard deviation
IQR: Interquartile range
OR: Odds ratios
95% CI: 95% confidence intervals
