# Supplementary material for: Differences in healthcare visit frequency and type one year prior to stroke among young versus middle-aged adults
Source: BMC Health Serv Res. 2021 Jan 22;21:84. doi: 10.1186/s12913-021-06064-5 (PMC7825199; doi:10.1186/s12913-021-06064-5)
Supplement: Supplementary file 1 — Additional file 1: Table 1. International Classification of Diseases, 9th Revision, Clinical Modification Codes Used to Identify Outcome or covariates of interest and MarketScan Procedure Group Codes. Table 2. Definitions used for classifying risk groups. [file 12913_2021_6064_MOESM1_ESM.docx]

| **Additional Table 1.** International Classification of Diseases, 9th Revision, Clinical Modification Codes Used to Identify Outcome or covariates of interest and MarketScan Procedure Group Codes | |
| --- | --- |
| **Outcome and Prior Medical History** | **ICD-9 Diagnostic Codes** |
| Stroke | 433.x1, 434 (excluding 434.x0 with exception of 434.00), 431, 436 |
| Diabetes mellitus | 250.xx, 648.0x |
| Hypertension | 401.x, 402.x0, 403.x0, 404.x0, 405.x, 642.0x, 642.1x, 642.2x, 642.7x |
| Obesity | 278.0x, 649.1x, V853, V854 |
| Influenza like illness | 079.89, 079.99, 460, 462, 464.xx, 465.x, 466.1, 466.19, 478.9, 480, 487.x, 490, 780.6, 784.1, 786.2 |
| Respiratory infection | 011, 012, 0.21.2, 022.1, 032, 033.x, 034.0, 039.1, 052.1, 055.1, 073.0, 112.4, 114.0, 114.4, 114.5, 115.x5, 130.4, 136.3, 460, 480.x, 481, 484, 513.0, 517.1, 784.91, 482.xx, 483.x, 484.x, 461.x, 462, 464.xx, 465.x, 485, 473.x, 480.x, 513.0, 517.1, 784.91 |
| GI Infections | 001.x, 002.x, 003.xx, 004.x, 005.xx, 006.x, 007.x, 008.xx, 009.x |
| Sepsis | 022.3, 036.2, 038.xx 054.5, 449, 670.2x, , 670.3x, 790.7, 995.91, 785.52 |
| Sexually Transmitted Infections | 054.xx, 091.xx, 092.x, 093.xx, 094.xx, 095.x, 096, 097.x, 098.xx, 099.xx, 131.xx, 647.0x, 647.1x, 647.2x |
| Urinary Tract Infection/ Genitourinary | 370.8, 590.xx, 595.0, 595.9, 597.xx, 598.xx, 599.0, 658.4, 658.43, 670.0x, 670.1x, 670.8x |
| Mastitis | 675.xx |
| Chorioamnionitis | 658.4x |
| Other infections | 020.x, 031.0, 083.0, 088.81, 114.1, 647.3x, 647.4x, 647.5x, 647.6x, 647.7x, 647.8x, 647.9x, 675, 658.4, 670, 672, 646.6, 681.xx, 682.x, 686.xx |
| Coagulopathy/ Hypercoagulable state | 286.xx, 287.1, 287.3, 2873, 287.4, 287.5, 289.81, 289.82, 649.3x |
| Nephrotic syndrome | 791.0, 581.81 |
| Chronic Renal disease | 581, 582, 583, 585, 587, 588, 646.2x |
| Sickle cell anemia | 282.4x, 282.6x |
| Migraine | 346.xx, 625.4, |
| Valvular disease | 093.2, 394.x, 395.x, 396.x, 397.x, 424.xx, |
| Congenital heart disease | 745.0, 745.1x,745.2, 745.3, 745.4, 745.5, 648.5x |
| Patent foramen ovale | 745.5 |
| Alcohol abuse | 291.xx, 303.xx, 305.0x |
| Drug dependency | 304.xx, 305.xx (except for 305.1), 648.3x, |
| Active smoking | 305.1x, 649.0 |
| Chronic ischemia | 412, 413.x, 414.xx |
| Congestive Heart Failure | 428.x2, 428.x3 |
| intracerebral hemorrhage | 431 |
| subarachnoid hemorrhage, | 430 |
| subdural or epidural hemorrhage, | 432 |
| pituitary apoplexy, cerebral venous thrombosis, | 253.2 |
| transient cerebral ischemia, | 435 |
| cerebral artery dissection | 443.21 , 443.24 |
| acute cerebrovascular disorder | 437.x |
| Trauma | 800 , 801, 802, 803, 804, 850, 851, 852, 853, 854 |
| **Visit Procedure Category** | **MarketScan Procedure Group Codes** |
| Preventive care | 115, 133 |
| Vaccination | 130 |
| Cardiovascular | 38, 39, 155, 156, 157, 160, 161, 163, 243 |
| Neurology | 74, 75, 175, 176, 177 |
| Chiropractic | 195, 191 |
| Emergent office | 110 |
|  |  |

| **Additional Table 2.** Definitions used for classifying risk groups. | |
| --- | --- |
| **Risk group** | **Definition** |
| Metabolic Syndrome | diabetes, hypertension and/or obesity |
| Infections | influenza-like illness, respiratory infection, gastro-intestinal infection, sepsis, sexually transmitted diseases, genitourinary infection, urinary tract infection, mastitis, chorioamnionitis and/or other infections |
| SITY risk factors | hypercoagulable state, coagulopathy, nephrotic syndrome, chronic renal disease, sickle cell anemia, migraine, valvular heart disease, congenital heart disease and/or patent foramen ovale |
| Substance Use | alcohol abuse, drug dependency or abuse, and/or current smoking |
| Vascular Disease | chronic ischemia, congestive heart failure, intracerebral hemorrhage, subarachnoid hemorrhage, subdural or epidural hemorrhage, pituitary apoplexy, cerebral venous thrombosis, transient cerebral ischemia, cerebral artery dissection and/or other acute cerebrovascular disorders |
| Trauma or Cancer | Trauma and/or cancer |
